# Graphene-Based Electrochemical Sensor for Detection of Hepatocellular Carcinoma Markers

**DOI:** 10.3389/fchem.2022.883627

**Published:** 2022-04-08

**Authors:** Ying Liang, Yuan Xu, Yaoyao Tong, Yue Chen, Xilu Chen, Shimin Wu

**Affiliations:** ^1^ Center for Clinical Laboratory, General Hospital of the Yangtze River Shipping, Wuhan Brain Hospital, Wuhan, China; ^2^ Center for Clinical Laboratory, Wuhan Hospital of Chinese Medicine, Wuhan, China

**Keywords:** hepatocellular carcinoma, tumor markers, electrochemical sensor, nanometer material, graphene

## Abstract

Hepatocellular carcinoma (HCC) is a group of highly lethal malignant tumors that seriously threaten human health. The main way to improve the survival quality and reduce the mortality of HCC is early diagnosis and treatment. Therefore, it will be of great significance to explore new quantitative detection methods for HCC markers. With the rapid development of electrochemical biosensors and nanomaterials, electrochemical sensors based on graphene can detect tumor markers, with the advantages of simple operation, high detection sensitivity, and specificity. Combined with the published literature in recent years, the article briefly reviews the application of graphene-based electrochemical biosensors in the detection of HCC markers, including alpha-fetoprotein (AFP), Golgi protein-73 (GP73), exosomes, and microRNA-122 (miR-122).

## Introduction

HCC is the sixth most commonly occurring cancer worldwide, and the mortality is second only to lung and gastric cancers (Altekruse, et al., 2009) (Bolhuis, et al., 2020). According to survey statistics, there are more than 700,000 new cases of HCC every year in the world (Qiao, et al., 2020). Because the clinical symptoms of HCC in the early stage are not typical, most patients are already in the middle or late stages when they are diagnosed (Al Bandar and Kim 2017). It is a need to explore new HCC surveillance tools for better detection and prevention with HCC.

Tumor markers are a series of substances that are produced by tumor cells themselves or by the surroundings, reflecting the existence and growth of tumors (Bohunicky and Mousa 2010) (Wu and Qu 2015). Tumor markers include DNA (such as specific mutations, translocations, amplification, and loss of heterozygosity) and different RNA or proteins (such as hormones, antibodies, oncogenes, or tumor suppressors). They exist widely in blood, body fluids, and tissues, which have a great value in tumor diagnosis and prognostic, evaluation of curative effect, and screening the high-risk populations (Yang and Patel 2019). Currently, the clinical detection of HCC markers mainly relies on enzyme-labeled immunoassay, chemiluminescence analysis, time-resolved fluorescence immunoassay, and protein chip detection (Wei et al., 2011) (Shen, et al., 2020). However, these methods are strict to reaction conditions and have low sensitivity. They also have the disadvantages of complicated operation and high cost that limit their further clinical application (Wiedemann 2013) (Wang et al., 2016) (Yang et al., 2019). Electrochemical sensor devices have been gradually applied to the quantitative analysis for tumor markers due to the high sensitivity and simplicity. With the progress and development of nanotechnology, nanomaterials based on electrochemical sensors have attracted a great deal of attention (Mohd Azmi et al., 2014). Among them, graphene is widely used due to the unique advantages, such as high mechanical strength, huge surface area, excellent electrical and thermal conductivity, high thermal stability, and good biocompatibility (Schedin et al., 2007). Graphene is monatomic-thickness-sheet nanomaterial, which is composed of Sp2 hybrid carbon atoms arranged in a honeycomb lattice structure. The unique electronic structure gives graphene a larger surface area and porous structure, which is suitable for multifunctional modification (Yoo, et al., 2008; Lawal 2015). In addition, graphene also has unique electrochemical properties such as fast electron transfer rate and low charge transfer resistance and excellent electrical and thermal conductivity, which improve the response speed and detection sensitivity of biosensors. Combining the advantages of both graphene structural and electrochemical properties, the graphene-based electrochemical biosensors have been applied for a broad range, including the detection of glucose, heavy metal ions, hydrogen peroxide, adenosine triphosphate, cholesterol, ascorbic acid, uric acid, pathogenic microorganisms, and cancer biomarkers (Shao, et al., 2010; Wang B. et al., 2017; Krishnan, et al., 2019; Sakar, et al., 2019; Taniselass, et al., 2019). This article briefly reviews the latest findings and progresses reported in the literature on the application of graphene-based electrochemical biosensors in the detection of HCC markers, including alpha-fetoprotein (AFP), Golgi protein-73 (GP73), exosomes, and microRNA-122 (miR-122).

## Alpha-Fetoprotein Detection

AFP is a plasma protein produced by the yolk sac and liver during the fetal period and is one of the most widely used clinical cancer markers, especially for liver cancer (Okuyucu, et al., 2015; Zhu, et al., 2015). AFP is produced in the fetal liver, with little or no production in normal tissues. When liver cells become cancerous, the gene expression of AFP is upregulated due to hypomethylation, and the concentration of AFP can be significantly increased in serum (Zhang, et al., 2018). Therefore, AFP becomes a common serum marker for early diagnosis of HCC (Zhao Y. et al., 2020). Many authoritative experts and organizations have designated AFP as the only tumor marker for primary liver cancer. The level of AFP in blood is related to the size of HCC tumor (Zhao B. et al., 2020). AFP detection has an important clinical value in the prognosis of surgical treatment, radiotherapy, and chemotherapy.

Graphene/nanoparticle composite-based electrochemical biosensors for the detection of AFP have been summarized in Table 1. Among them, the graphene–AuNPs composite exhibited outstanding electrocatalytic activity, which combines the advantages of high electrical conductivity and electrocatalytic activity of AuNPs. For example, Zhou et al. (2019) synthesized and immobilized gold nanoparticles–dextran–reduced graphene oxide (AuNPs–Dex–rGO) onto an electrode, with ferricyanide as the electrochemical probe, and the simple electrochemical immunosensor achieved ultrasensitive detection of AFP. A significant advantage of this method is that the AuNPs–Dex–rGO multifunctional nanocomposites were prepared by a simple one-step process, which can greatly simplify the steps and ensure synergistic effect. In addition, due to the excellent conductivity and material compatibility of rGO and AuNPs, the electron transfer efficiency of the sensor was greatly improved, exhibiting high sensitivity and low detection limit (0.05 pg/ml). Shan et al. (2016) prepared a kind of novel redox-active species poly(MB)-Au using HAuCl_4_ as an oxidant, and methylene blue as a monomer. Then, they immobilized the antibody on the nanocomposites as a probe and modified graphene–Au as the substrate of the electrode. They pioneered the synthesis of uniform poly (methylene blue)–Au nanoparticles in an approach of dye oxidative polymerized by noble metal salt. Due to the uniform and conductive poly (methylene blue)–Au nanomaterials with an innate signal used as immunosensing probes, the sensor improved signal amplification and provided a detection limit of AFP with 0.0196 pg/ml. Furthermore, because this assay selected the sandwich method mode, which owns high detection efficiency, the detection results of this method on clinical serum were well consistent compared to those ofs ELISA, showing good prospects for clinical application.

**TABLE 1:** Graphene nanocomposite-based electrochemical assay for AFP.

Graphene-based nanocomposite	Linear range	LOD	Reference
AuNPs–Dex–rGO	0.01–20 pg/ml	0.05 pg/ml	Zhou et al. (2019)
Graphene–Au	1 pg/ml–100 ng/ml	0.0196 pg/ml	Shan et al. (2016)
PtNPs/Co_3_O_4_/graphene	0.1 pg/ml–60 ng/ml	0.029 pg/ml	Liu et al. (2017)
TB–Au–Fe_3_O_4_–rGO	0.01 pg/ml–10 ng/ml	0.0027 pg/ml	Wang Y. et al. (2017)
CdTe@SiO_2_/graphene	1.0 pg/ml–100 ng/ml	0.22 pg/ml	Pan et al. (2019)
AuNPs/PGNR	5–60 ng/ml	1 ng/ml	Jothi et al. (2020)

Graphene and metal oxide composites are the current focus. The combination of Fe_3_O_4_ and reduced graphene oxide (rGO) improves not only the surface area for the immobilized antibody but also the electrocatalytic ability. Liu et al. (2017) reported a sandwich electrochemical biosensor for AFP based on the signal amplification of Pt NPs/Co_3_O_4_/graphene-modified secondary antibodies. Under optimal conditions, the sensor allowed linear detection of AFP in the range of 0.0001 ng/ml–60 ng/ml with a detection limit of 0.029 pg/ml. The sensor could be also stored for a long time at 4°C, showing outstanding stability. Wang Y. et al. (2017) demonstrated a biosensor using highly conductive graphene nanocomposites by immobilizing gold nanoparticles and toluidine blue (TB) on Fe_3_O_4_–rGO nanocomposite, especially the TB–Au–Fe_3_O_4_–rGO nanocomposite was fabricated using Fe_3_O_4_–rGO as a single-layer and high-purity platform to absorb gold nanoparticles (AuNPs) and TB. AuNPs have good biocompatibility in capturing antibodies, and Fe_3_O_4_ NPs have good electrocatalytic performance toward TB, which was detected as a redox probe. So, the usage of the TB–Au–Fe_3_O_4_–rGO-modified electrode to fabricate the electrochemical immunosensor can achieve the quantitative detection of AFP. Due to the large specific surface area and high electrical conductivity of graphene, the TB–Au–Fe_3_O_4_–rGO-modified electrode has good adsorption property and can load a large number of TB and Au, which greatly achieve the effective and sensitive detection of AFP. Under optimal conditions, the method exhibited a low detection limit of 0.0027 pg/ml for AFP. As a successful biosensor with outstanding detection capability, the method makes full use of the advantages of elements composited of the multifunctionalized nanocomposites, including graphene, Fe_3_O_4_ NPs, AuNPs, and TB.

In addition, some novel nanocomposites and skillful chemical reduction methods were also explored in the application of AFP detection. For example, Pan et al. (2019) constructed a sandwiched immunosensor of AFP using CdTe@SiO_2_ as an emitter, proving a low detection limit of 0.22 pg/ml. However, the distinctive degrees of toxicity manifested by QDs still remain to be addressed. Jothi et al. (2020) made AuNPs electrodeposited on porous graphene nanoribbon by the chemical reduction method to act as a platform to detect AFP, with a low detection limit of 1 ng/ml, providing versatile detection strategies.

## Golgi Protein-73 Detection

GP73 is a 400-amino acid protein with a predicted molecular weight of 73 kDa (Duc, et al., 2020). As a kind of transmembrane protein, GP73 mainly exists on the surface of the Golgi apparatus (Kladney, et al., 2000). However, when hepatocytes become cancerous, endoprotease cleaves, and GP73 is released into the extracellular environment; therefore, serotype GP73 can be detected in blood serum of liver cancer patients. GP73 in liver cancer tissue is positively correlated with vascular endothelial growth factor (Mao, et al., 2010). Related studies also show that serum GP73 can be used to evaluate the prognosis of liver cancer (Zhao S. et al., 2020).

Yu, et al. (2014) reported an electrochemical immunosensor for detecting GP73 with the combination of graphene oxide and CdSe QDs. rGO provides excellent conductivity, and the CdSe QDs are detected with a prominent electrochemical signal. The sensor showed a linear range from 20 to 5,000 pM and sensitivity of 12 pM for GP73 detection. As short strands of DNA or RNA, aptamers are often used as recognition elements for marker detection due to the high affinity and specific for the target (Keefe, et al., 2010). Compared with the traditional biomolecular ligands such as antibodies, aptamers have other merits of good stability, lower cost, and easy modification. An example of the aptamer-based biosensor is discussed here. Zhou et al. (2020) found that the hemin/graphene nanohybrids (HGNs) can catalyze silver deposition to amplify the signal. They solidified GP73 aptamer as the capture probe, and assembled HGNs–aptamer (HGNs–Apt) as the signal probe. Then, the silver (Ag) ions were reduced in solution due to the peroxidase-like properties of HGNs. The electrochemical aptamer nanobiosensor allows linear detection of GP73 between 10.0 and 100.0 μg/ml with a detection limit of 3.16 μg/ml.

## Exosomes Detection

Exosomes are nanoscale bilayer lipid inclusion structures with a diameter of 50–200 nm, carrying biological information of maternal cell molecule characteristics, such as proteins, lipids, deoxyribonucleic acid (DNA), messenger RNA (mRNA), microRNA (miRNA), and noncoding RNA (Iero, et al., 2008). Exosomes widely exist in body fluids, including blood, tears, urine, saliva, and ascites. Studies have shown that tumor cells can release more exosomes than normal cells, and the basic information of cancer cells can be directly obtained by analyzing exosomes (An, et al., 2015). Exosomes are involved in various processes of tumor occurrence and development, including promoting tumor growth, metastasis, and drug resistance and causing the changes of the tumor microenvironment (Webber, et al., 2015).

Exosomes contain a large number and variety of biologically active contents, which can be detected as biomarkers and potential therapeutic targets (Greening, et al., 2015). An et al. (2019) reported a click chemistry and DNA hybridization chain reaction (HCR)-based electrochemical sensor for detecting tumor exosomes. They immobilized the CD63 aptamer on the graphene-modified electrode to capture exosomes. Based on the click chemistry method, the captured exosomes were labeled with a DNA probe, and DNA HCR was triggered to bind numerous of horseradish peroxidase (HRP) molecules. The concentration of exosomes was quantified by monitoring the electrochemical current of HRP molecules catalyzing the reaction of o-phenylenediamine (OPD) and H_2_O_2_. For the ultrasensitive detection of tumor exosomes, they flexibly used alkynyl-4-ONE for the conjugation of protein on the surface of exosomes to detect other exosome subpopulations. Meanwhile, the DNA HCR was also designed for signal amplification. This method can linearly detect exosomes from 1.12 × 10^2^ to 1.12 × 10^8^ particles/μl with detection limits up to 96 particles/μl. Giving its biocompatibility associated with high stability, the method enabled sensitive and accurate analysis of exosomes in human serum.

Hepatoma-derived exosomes can release vascular endothelial growth factor (VEGF), which can promote tumor angiogenesis, provide sufficient nutrition for HCC, and promote the occurrence and development of liver cancer cells (Mosquera-Heredia and Maria, 2021). Ni et al. (2020) synthesized methylene blue-loaded graphene oxide (GO/MB) composite and modified ferrocene-labeled aptamer onto glassy carbon (GC) to develop an aptasensor for the detection of vascular endothelial growth factor (VEGF). The GC–GO/MB–streptavidin aptamer acts as a sensitive redox probe and is capable of amplifying electrochemical signals, with a linear detection range of 10–500 pg/ml for VEGF.

## MicroRNA-122 Detection

MicroRNAs (miRNA) are a kind of small endogenous single-stranded noncoding RNAs, which can regulate the expression level of target genes at the posttranscriptional level (Hu, et al., 2012). MiR-122 is the most abundant miRNA molecule expressed in liver tissue, accounting for about 70% of the total miRNA in the liver level (Li, et al., 2019). Studies have shown that the expression of miR-122 decreased significantly in patients with liver cancer (Lewis and Jopling 2010). MiR-122 can inhibit the growth of liver cancer cells and prevent the formation of new blood vessels, by inhibiting the formation of vascular endothelial cells in the microenvironment of HCC (Bandiera, et al., 2015). These mechanisms reduce the nutrients required by liver cancer cells, which is not enough to maintain the normal demand and prevent the growth of liver cancer cells (Nassirpour, et al., 2013).

Kasturi et al. (2021) developed a biosensor using gold nanoparticles–dotted reduced graphene oxide (rGO/Au) nanocomposite for the detection of miR-122. A linear concentration with a range from 10 μM to 10 pM and a detection limit of 1.73 pM were obtained. Surprisingly, they synthesized rGO by a “green” approach with natural soapsuds instead of toxic chemicals such as NaBH_4_ and hydrazine like most researchers often do, providing us the precious environmental conscientiousness. Kilic et al. (2016) designed an electrochemical biosensor by first immobilizing the complementary anti-miR-122 and then capturing and solidifying the target miR-122. The advantage of this method is that it is less complicated and label-free. The proposed biosensor can detect the limit of 1 pM in real samples such as total RNAs isolated from cell lysates.

## Outlook

With the development of gene-sequencing technology in recent years, more and more tumor markers of HCC have been discovered and studied. Electrochemical technology is an interdisciplinary comprehensive technology and has been widely used in clinical laboratory and tumor detection. Compared with traditional detection methods in clinical HCC marker diagnosis, electrochemical immunosensors based on graphene have a series of advantages such as short detection time, good selectivity, low detection limit, and wide detection range. The future research from functional perspective needs more attention in following aspects including 1) new graphene nanocomposites with more stability in a high ionic strength environment and stronger ability of binding with biomolecules; 2) how to use one graphene sensor to realize the combined detection of multiple tumor markers simultaneously; 3) the novel tumor markers of liver cancer such as circRNA based on the graphene-assisted electrochemical sensor should be studied. From the perspective of real biological application, there are still several challenges related to practical production including 1) the method of mass production of single-layer sheet graphene has not been widely used; 2) in the process of modification and functionalization of large amounts of graphene, the stacking and agglomeration of the nanocomposite is hard to alleviate; 3) there is a great demand for the POCT system in clinical detection at present, and the electrochemical sensor based on graphene must develop and transfer to the miniaturization and intelligence of electronic devices; 4) there is a possibility that the graphene-based electrochemical sensor can be reasonably combined with some new assay technologies, such as chromatography and sequencing, which may make the whole detection process develop toward intelligence and automation. These advances in the research of the graphene-based electrochemical sensor provide new promising platforms for sensitive detection of HCC markers, which provide potential solutions not only in biomedical assay but also in other fields such as food, agriculture, environmental monitoring, and control.
