# Corrigendum: Graphene-Based Electrochemical Sensor for Detection of Hepatocellular Carcinoma Markers

**DOI:** 10.3389/fchem.2022.917820

**Published:** 2022-04-28

**Authors:** Ying Liang, Yuan Xu, Yaoyao Tong, Yue Chen, Xilu Chen, Shimin Wu

**Affiliations:** ^1^ Center for Clinical Laboratory, General Hospital of the Yangtze River Shipping, Wuhan Brain Hospital, Wuhan, China; ^2^ Center for Clinical Laboratory, Wuhan Hospital of Chinese Medicine, Wuhan, China

**Keywords:** hepatocellular carcinoma, tumor markers, electrochemical sensor, nanometer material, graphene

In the original article, there was a mistake in the order of the listed authors. The correct order is “Ying Liang, Yuan Xu, Yaoyao Tong, Yue Chen, Xilu Chen and Shimin Wu”.

The authors apologize for this error and state that this does not change the scientific conclusions of the article in any way. The original article has been updated.

